# Multivariate Statistical Optimization of a Modified Protocol of the Ionic Polyelectrolyte Pre-Gelation Method to Synthesize Alginate–Chitosan-Based Nanoparticles

**DOI:** 10.3390/polym18010077

**Published:** 2025-12-26

**Authors:** Ángela J. Rodríguez-Talavera, Sara Gálvez-Rodríguez, Juan M. Rodríguez-Díaz, Edgar Pérez-Herrero

**Affiliations:** 1 Grupo de Investigación de Encapsulación y Evaluación Biológica Avanzada (ENCAPBIO-ULL Research Group), Departamento de Ingeniería Química y Tecnología Farmacéutica, Universidad de La Laguna, Avenida Astrofísico Francisco Sánchez, 38206 La Laguna (Santa Cruz de Tenerife), Spain; 2 Escuela de Doctorado y Estudios de Posgrado, Universidad de La Laguna, Avenida Astrofísico Francisco Sánchez, 38206 La Laguna (Santa Cruz de Tenerife), Spain; 3 Departamento de Estadística/Instituto Universitario de Física Fundamental y Matemáticas, Universidad de Salamanca, 37008 Salamanca, Spain

**Keywords:** alginate, chitosan, polymer biomaterial, nanoparticles, drug delivery system, mathematical model, factorial design

## Abstract

Alginate [ALG] and chitosan [CS] are biomaterials of importance in drug-delivery because of their ability to form ionically-crosslinked nanosystems and polyelectrolyte-complexes under mild conditions. Here, a modified ionic-polyelectrolyte-pre-gelation method, with a controlled addition of reagents and sonication throughout the process, is reported to produce ALG¬¬-CS-based NPs. A mathematical study of the effects of the factors with influence in the process on the properties of NPs has been performed using a two-phase-design-of-experiment-based procedure, something never done to our knowledge. The concentration of ALG, CS and CaCl_2_ and the ratio ALG:CS have significant influence on polydispersity (PDI), surface-charge (ZP) and encapsulation efficiency (EE%) of NPs. Moreover, CS flow rate has a significant effect over PDI and EE%. Thus, the values of ALG, CS and CaCl_2_ concentration (mg/mL), ALG:CS (mL:mL) and CS flow rate (mL/min) to obtain the minimum-expected PDI (0.168) or the optimized EE% (86.7) are {0.30, 0.79, 1.00, 2.50:1.00, 0.86} or {0.30, 1.00, 1.00, 2.50:1.00, 2.00}, with ALG:CaCl_2_ (mL:mL) and CaCl_2_ flow rate (mL/min) fixed at 2.50:0.31 and 1.25. Although most of the conditions yielded highly-negative particles (minimum-expected of −67.8 mV using 0.69, 0.30 and 0.13 mg/mL of ALG, CS and CaCl_2_ and ALG:CS of 2.50:0.59 mL:mL), varying the mass ratio of ALG:CS allowed for the generation of positively-charged particles (up to +21.1 mV with 0.30, 1.00 and 0.61 mg/mL of ALG, CS and CaCl_2_ and ALG:CS of 2.50:1.00 mL:mL). In both cases, ALG:CaCl_2_ (mL:mL), CaCl_2_ and CS flow rates (mL/min) were fixed at 2.50:0.31, 1.25 and 1.25. The model for the NPs size depends only on CS and CaCl_2_ concentrations, with the minimum- or maximum-expected (160 or 635 nm) at 0.30 and 1.00 or 1.00 and 0.30 of CS and CaCl_2_, although the method allows a wide range of sizes (144.0–1965.0 nm).

## 1. Introduction

The use of natural biopolymers, mainly polysaccharides like alginate (ALG) and chitosan (CS), in the formulation of drug delivery systems is a practice commonly addressed by the scientific community since these biomaterials can be easily obtained from natural sources, showing low immunogenicity and toxicity and excellent biocompatibility/biodegradability, and, more importantly, they allow the synthesis of nanocarriers by mild-based techniques that avoid the use of organic solvents and high temperatures [1,2,3,4]. In this regard, the ionic gelation-based methods easily generate ionically crosslinked nanosystems by the simple interaction of the carboxyl groups of ALG or the amine groups of CS with counterions, such as calcium chloride (CaCl_2_) or sodium tripolyphosphate (TPP), respectively [2,5,6]. Furthermore, the combination of the functional groups of ALG and CS allows the generation of polyelectrolyte complexes [7,8]. The incorporation of CS to ALG-based nanoparticles has been reported to slow down the drug release from the porous ALG matrix and stabilize and reinforce the nanosystem [2,9]. In any case, many authors have demonstrated the convenience of incorporating both biomaterials, ALG and CS, in the formulation of drug nanocarriers to take advantage of the synergies generated between both materials in terms of entrapment efficiency and controlled drug release [2,5,6,10].

Some authors have described two-step synthesis processes to prepare ALG–CS-based nanoparticles (ALG-CS NPs) by means of the ionic polyelectrolyte pre-gelation technique, which is mainly based on the generation of ALG pregels with Ca^2+^ ions (CaCl_2_) that are further stabilized with CS to form polyelectrolyte complexes [5,6,7,9,11,12,13,14,15,16]. This methodology, which was first described in 1993 by the group of Prof. Couvreur [17], using poly-L-lysine as polycation, has evolved to enhance the properties of the nanoparticles and adapt the formulations to entrap different therapeutic molecules. However, a complete mathematical analysis to study how all the variables that are involved in the process of the synthesis affect their main characteristics (size, polydispersity and surface charge) has not yet been performed. Still, some authors have reported statistical studies to partially optimize this synthesis process. Thus, Kushwaha & Dwivedi [18] performed a 3^2^ factorial design to observe the effect of ALG and CS concentrations on the *mean particle size*, *surface charge* (*ZP*) and *encapsulation efficiency* (*EE*%). Later, Zohri et al. [12] studied the effect of the CS:ALG ratio, the pH of CS and the stirring rate on the *mean size*, *polydispersity* (*PDI*), *ZP*, *EE*%, cumulative release and morphological degradation time of the particles, using a response surface methodology with a Box–Behnken design. Similarly, Kohli et al. [13] used a Box–Behnken statistical analysis to investigate the effect of three variables (CS, ALG and CaCl_2_ concentrations) on *mean size*, *PDI* and *ZP* of the nanoparticles generated.

In this work, for the first time, a two-step design of experiments (DoE) methodology has been carried out to detect all the factors that can influence the ionic polyelectrolyte pre-gelation method to generate ALG–CS NPs, which was enhanced by introducing the controlled addition of reagents and sonication throughout the process to improve mixing of phases. Statistical methodology consists of an initial screening to remove the non-influential factors and a subsequent response surface methodology (RSM) to find the dependence of multiple response variables (*size*, *PDI*, *ZP* and *EE*%) on the influential factors of the synthesis process. The effects of these factors on the responses have been studied to provide the optimal conditions of synthesis (minimum PDI and maximum EE%) and the correlations between the influential factors and the responses *size* and *ZP*.

## 2. Materials and Methods

### 2.1. Materials

Alginate acid sodium salt (ALG) from brown algae (medium viscosity, ≥2000 cP, 2% at 25 °C), bovine serum albumin (BSA) and calcium chloride (CaCl_2_) were provided by Sigma-Aldrich (Merck KGaA, St. Louis, MO, USA). Chitosan lactate (CS) from crustaceans (low viscosity, <200 cP, 1% at 25 °C, degree of deacetylation ≤ 95%) was acquired from ChitoLytic (Toronto, ON, Canada). All solutions were prepared employing purified water. Bradford’s solution for protein determination was acquired from PanReac AppliChem (Barcelona, Spain).

### 2.2. Synthesis and Characterization of Alginate-Chitosan Nanoparticles (ALG-CS NPs)

**Figure 1:**
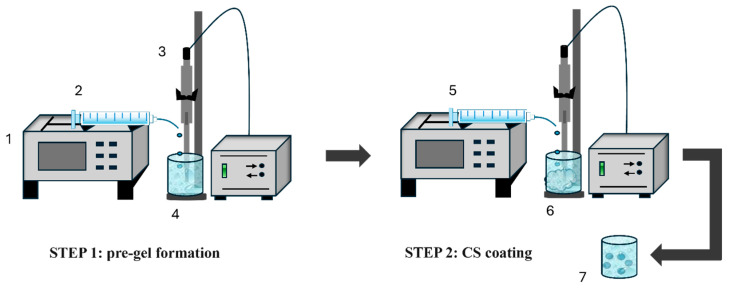
Procedure of synthesis of BSA-loaded ALG–CS NPs by the modified ionic polyelectrolyte pre-gelation method using sonication throughout the process and syringe pump-controlled flow rates. (1) Syringe pump; (2) CaCl_2_-BSA solution; (3) Sonicator; (4) ALG solution; (5) CS solution; (6) ALG pregel; (7) BSA-loaded ALG-CS NPs.

ALG–CS NPs were prepared in two steps by the ionic polyelectrolyte pre-gelation method described by Loquercio et al. [5], but with important modifications, such as the controlled addition of the reagents by means of a syringe pump and the use of sonication for mixing the phases during the whole procedure. Briefly, an ALG pregel was generated by adding an aqueous solution of CaCl_2_ (0.13–1.00 mg/mL, pH: 6.8, µ: 30 mM) to an acidic (pH 3.7) aqueous ALG solution (0.30–1.00 mg/mL, µ: 5 mM), under sonication (55 W, amplitude: 20%, Q55, QSONICA LLC, Newtown, CT, USA) and with a controlled flow rate from 0.50 to 2.00 mL/min, using a syringe pump (Legato 110, Kd Scientific, Holliston, MA, USA). Subsequently, an aqueous CS solution (0.30–1.00 mg/mL, pH: 4.0, µ: 3 mM) was added to the previously generated ALG pregel under sonication at different flow rates (0.50–2.00 mL/min) using a syringe pump. Apart from the reagent concentrations and flow rates, the volume ratios ALG:CS and ALG:CaCl_2_ were varied, using a fixed volume of ALG of 2.50 mL and varying the volumes of CS, from 0.25 to 1.00 mL, and CaCl_2_, from 0.16 to 0.46 mL. BSA-loaded ALG–CS NPs were prepared following the above process but adding the protein in the CaCl_2_ solution at a concentration ranging from 1.00 to 2.90 mg/mL. All ALG–CS NPs generated were isolated by centrifugation (19,000 RCF, 30 min). A scheme of the synthesis process is shown in Figure 1.

Once prepared, the ALG–CS NPs were characterized in terms of *size* and *polydispersity* (*polydispersity index*, *PDI*) by dynamic light scattering (DLS), and *surface charge* (*Z Potential*, *ZP*) by electrophoretic light scattering (ELS) (Zetasizer NanoZS, Malvern Panalytical, UK) in Servicio General de Apoyo a la Investigación of Universidad de La Laguna (SEGAI-ULL). The encapsulation efficiencies (*EE*%) of the nanoparticles were determined, using BSA as a model protein, with the Bradford protein assay, comparing the absorbance of each sample to a standard curve constructed by a set of protein standards, at 595 nm using a microplate reader (Infinite 200, Tecan Trading AG, Männedorf, Switzerland).

### 2.3. Experimental DoE Design and Statistical Analysis

A two-step DoE procedure was performed to detect the factors that may have influence on the synthesis process, estimate their effects and obtain the relation between factors and the set of responses (*mean size*, *PDI*, *ZP* and *EE*%), allowing the optimal synthesis conditions of the ALG–CS NPs to be obtained. Initially a screening approach was used to remove the non-influential factors, using two-level fractional factorial designs with maximal resolution to reduce the number of experiments and at the same time avoid the confusion of main factors with low-level interactions. Once the most important factors were identified, a response surface methodology (RSM) was performed to obtain second-order models that fit approximately the dependence of the response variables on the influential factors of the synthesis process. ANOVA methods were used to detect significant effects at the different stages of the procedure, using SPSS v.22 and Mathematica 13.2 software. The computation of the optimal conditions for maximizing/minimizing the responses was done by employing the corresponding RSM methods and numerical procedures with Mathematica.

## 3. Results and Discussion

### 3.1. Synthesis Approach to Obtain ALG-CS NPs by a Modified Ionic Polyelectrolyte Pregelation Method

The polyelectrolyte pre-gelation method permits to obtain ALG-CS-based NPs by using only three biocompatible reactants, ALG, CS and CaCl_2_, avoiding the organic solvents and the emulsification steps, which are commonly used in the synthesis of nanoparticles but can endanger the integrity of encapsulated bioactive molecules [5,7,13]. As mentioned above, the addition of the polyelectrolyte complexation with CS to the ionotropic gelation reaction allows for improved controlled drug delivery and strengthens the integrity of the ALG-based nanosystem [9]. The literature suggests that pre-gelation of ALG with CaCl_2_ is required for ionic interaction between CaCl_2_, ALG and CS to take place, which will result in stable nanoparticles [5,13]. Thus, considering the standard ionic polyelectrolyte pre-gelation method, concentrations and volume ratios of all the reagents (ALG, CS and CaCl_2_) can be identified as the main factors that can affect this synthesis process. Moreover, as far as we know to date, in this technique the addition of CaCl_2_ and CS has been done dropwise, without controlling flow rates, so the use of syringe pumps to do that seems to be an interesting improvement to the conventional methodology, being their flow rates two new variables to take into account. It should be noted that a controlled addition of reagents, with stable and reproducible flow rates, is reflected in a better mixing of phases with a tight control on the gelation process, resulting in more uniform and reproducible conditions [19,20]. According to these facts, seven factors were identified with possible influence in this synthetic process. After some preliminary tests and a deep literature review, the levels of these seven factors were set as distant as possible to be able to determine the significance of them but allowing the correct generation of the ALG–CS NPs (Table 1). Note that the general ranges for the levels were initially selected through a literature search, and were experimentally fixed considering all factors and using particle formation as the general criteria. Outside of the levels shown in Table 1, visible precipitates and/or aggregates in the micron range were obtained.

Furthermore, although sonication has been used by some authors in this procedure, it has generally been applied to the generated nanoparticles to avoid their aggregation, i.e., it is used after the mixing of the phases by magnetic stirring [5,7,13]. However, sonication throughout the synthesis process can improve mixing, both during pregel formation and in the subsequent polyelectrolyte complexation between ALG and CS, achieving more homogeneous and smaller particles [21]. Note that the local shear and cavitation generated by ultrasonication break the possible gelled aggregates and can influence the gelation reaction, reducing the hydrodynamic diameter of nanoparticles and narrowing their size distribution [22,23]. This claim was demonstrated by the values of *size* and *PDI* obtained when synthesizing ALG–CS NPs under ultrasonication or using magnetic stirring (Table 2). Therefore, sonication was included in both stages of the synthesis process, not adding this factor to the other seven already considered since it was fixed because it meant a significant improvement in the process of synthesis.

**Figure 2:**
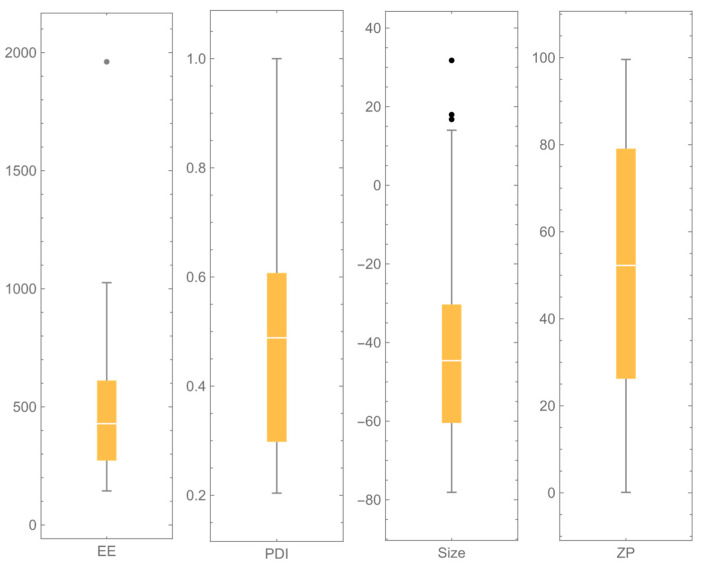
Boxwhisker charts of the four responses, where outliers are shown.

As can be seen in Table 3, the modification of the factors considered, at the contemplated levels, allowed for the obtaining of ALG–CS NPs of very different *mean sizes*, which ranged from 144.0 to 1965.0 nm. Some experimental conditions (e.g., low concentration of ALG) permitted to achieve *PDI* values below 0.4, but, in most cases, this acceptable range of values was not obtained, so polydispersity should be a response to being minimized. The values of *ZP* showed that the surface charge of nanoparticles was mostly highly negative due to the carboxylic groups of ALG, except in cases where the proportion of CS was much higher than ALG. Regarding *EE*%, the values obtained were very much dependent on the levels of the factors, but reaching values as high as 99.6%, making this another response to be optimized, taking into account the values of the rest of the responses. The dispersion graphs of the four response variables are shown in Figure 2.

### 3.2. Two-Step DoE Statistical Analysis and Optimization

#### 3.2.1. Screening Methodology

The factors with influence on the response variables were determined by a screening procedure using a main-effect model, i.e., containing just the effects of the seven factors and the constant term (eight parameters). A Plackett–Burman design was initially considered, but, after a thorough revision of the specialized literature, it was discarded to avoid confusion between the main effects and the two-factor interactions [24]. A 2_IV_^7-3^ fractional design, with maximum resolution and minimum aberration, was chosen, considering only 2 extreme values for each factor, −1 (low level) and +1 (high level), to reduce the number of experiments. When using this design, the main effects would be confounded, in the worst scenario, with three-factor or higher interactions (resolution 4) and the number of low-order interactions confounded is minimized. The complete defining relation is given at the end of Appendix A. Table 4 shows the design matrix for the experiments and the values of the responses obtained in this step, highlighting that experiments were performed by two operators in a random order (although, when using these observations, it was checked that the operator had no effect on the responses). For the detailed results of the screening procedure see Appendix A.

In order to use all the available information, apart from data from Table 4, the preliminary experiments that were performed at the final levels of the factors (Table 3) were also considered in the screening procedure. It should be noted that many preliminary experiments were done to define the actual levels for the factors (assuring the correct generation of the ALG–CS NPs), with only those experiments that were performed at the final levels of the factors (as stated in Table 1) being shown in Table 3. The preliminary experiments of Table 3 were conducted under the same conditions as the ones obtained from the 2_IV_^7-3^ fractional design of Table 4. When preliminary experiments were considered in the analysis, the results found the operator’s influence significant in two of the responses, reducing the unexplained variability. Therefore, it is possible to talk about a ‘training effect’ since the operator’s influence is observed just in the preliminary experiments performed to define the factor levels, but not in the experiments performed later, designed to evaluate the factor’s influence and fitting the models for the responses.

Results indicated that the four first factors (ALG, CS and CaCl_2_ concentrations and ALG:CS volume ratio) seem to have influence in at least one response variable, while ALG:CaCl_2_ volume ratio and CaCl_2_ flow rate show very little importance, and CS flow rate does not provide clear conclusions (see Appendix A. Detailed Results of the Screening Procedure). It is important to highlight that the last two factors (CaCl_2_ and CS flow rates) were always observed at a constant level in the preliminary experiments (Table 3), so those experiments do not provide information about how the variation of the two factors may affect responses, maintaining uncertainty in the model about CS flow rate. Therefore, for all this, the reasonable proposal is the removal of factors 5 (ALG:CaCl_2_ volume ratio) and 6 (CaCl_2_ flow rate).

#### 3.2.2. Response Surface Methodology

After identifying the factors with influence in the modified ionic polyelectrolyte pre-gelation synthesis method, the non-influential ones were removed from the model, which in the experimental language means that from this moment on the following experiments were performed for fixed values of these factors. A Response Surface Methodology (RSM) was applied to the remaining five factors to obtain approximate models for all the response variables (*mean size*, *PDI*, *ZP* and *EE*%). A new central level, “0” in coded variables, was added for every factor to use central and axial observations. In natural variables, this approach means taking observations at the mean of the extreme levels of the factor. Table 5 includes the new configuration of the levels of the factors, where the removed factors are fixed at the corresponding central level in the forthcoming experiments.

As previously mentioned, when fixing the levels of factors, those were taken as distant as possible, as long as they allowed for proper generation of nanoparticles, so the experimental region is cuboidal. Initially, a first order model was proposed to perform the Steepest Ascent Method, but the existence of curvature discarded this simple model. Then, full quadratic models were fitted to all the response variables using the least squares method, for which central and axial points in the faces of the hypercube were added to the full factorial design to create a non-rotatable Central Composite Design (CCD) for the procedure. Note that, when possible, rotatable designs are preferred since for these designs the variance of the prediction of the responses at a certain point (for specific conditions of the factors) only depends on the point’s distance to the center, that is, this variance is constant on spheres. However, to achieve this the nonzero coordinate of the axial points should be 2^5/4^ > 1, which means that the observations should be taken out of the limits of the experimental region, and that is not possible since the limits of this region have been fixed at the extreme feasible values of the factors for the particle to be formed. Table 6 and Table 7 include the CCD design matrix, as well as the values of the responses for all these experiments, including Table 6 the 32 rows from the 2^5^ factorial design and Table 7 the central points (5 rows) and the axial points (the last 10 rows). All the experiments were carried out in a random order.

Below are the results of the application of the RSM-based procedure to the modified ionic polyelectrolyte pre-gelation synthesis method for all the response variables considered in this study. Model and parameter statistics are shown in Appendix B (Model Statistics for RSM Models), together with fitting statistics of the final model-proposals, the standard error prediction at the optimal conditions for each response, and for each one a graph of residuals vs. predicted values, which do not show any particular pattern.

Model for the response variable “*EE*%”.

**Figure 3:**
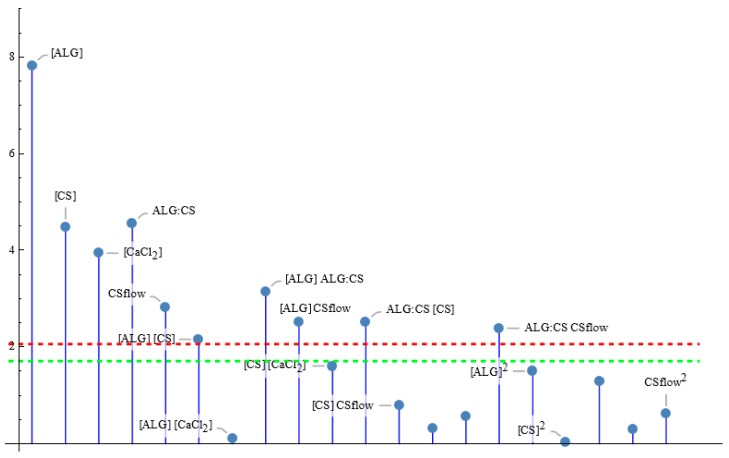
Parameter *t*-test absolute values for the *EE*% model. Values over the green line are significant at 10% significance level, those above the red line are significant for 5% level of significance.

One of the main characteristics that any formulation must have is the possibility of incorporating a drug to transport it to the therapeutic target and generate its controlled release there. Therefore, it is very important to find the conditions that maximize *EE*% in the ALG–CS NPs prepared by the synthesis approach reported in this work, taking into account its loading capacity. Figure 3 plots the *p*-values of the tests for the model parameters with effect in this modified synthesis procedure, showing that the significant factors are the concentration of all the reactants (‘[ALG]’, ‘[CS]’ and ‘[CaCl_2_]’), the ALG:CS volume ratio (‘ALG:CS’) and the CS flow rate (‘CSflow’), as well as some interaction terms of the main effects. Then, the final fitted model for EE% is stated in Equation (1) that depends on the five factors through the five main effects and five two-factor interactions.

*EE*% = −62.655 + 20.027 [CaCl_2_] + 27.192 [CS] + 34.566 CSflow + 37.973 [ALG] + 58.241 ALG:CS −19.333 [ALG:CS] CSflow − 21.762 [ALG] CSflow − 40.102 [ALG] [CS] + 43.524 ALG:CS [CS] −54.667 [ALG] ALG:CS(1)

where ‘[ALG]’ refers to ALG concentration, ‘[CS]’ to CS concentration, ‘[CaCl_2_]’ to CaCl_2_ concentration, ‘ALG:CS’ to ALG:CS volume ratio and ‘CSflow’ to CS flow rate.

**Figure 4:**
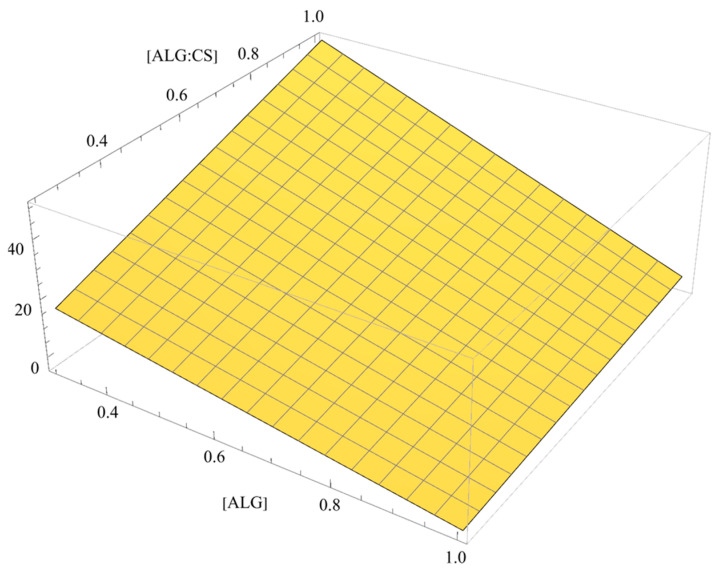
*EE*% as a function of [ALG] and ALG:CS volume ratio for central values of the rest of factors.

Considering that the proposed model here is not valid out of the borders of the experimental region, the conditions that maximize the *EE*% are attained in some of the vertices of the cuboid. Thus, the maximum value expected for EE% (86.7%) can be achieved for the five factor values {−1, 1, 1, 1, 1}, in coded variables, i.e., [ALG] = 0.30 mg/mL; [CS] = 1.00 mg/mL; [CaCl_2_] = 1.00 mg/mL; ALG:CS volume ratio = 2.50:1.00 mL:mL (molar ratio = 1.0:1.0 mol:mol); CS flow rate = 2.00 mL/min, in natural variables, the rest being fixed at the central levels. The most influential factors seem to be [ALG] and ALG:CS volume ratio, that participate in every interaction, including their own; Figure 4 shows the response as a function of the two factors assuming constant (central) values for the rest of the factors. Moreover, it should be noted that BSA is incorporated in the CaCl_2_ solution (pH: 6.8), as the model drug, which implies that the protein behaves as a negatively charged molecule (pI: 4.7), potentially competing with ALG for CS reacting groups and slightly altering the ALG:CS optimal ratio.

Model for the response variable “*PDI*”.

Obtaining monodisperse nanoparticles is crucial for their application as drug delivery systems since it assures the stability and control of the formulation, as well as its reproducibility batch to batch, and permits a consistent controlled drug release and predictable interaction with biological systems. To obtain monodisperse nanocarriers, *PDI* should be minimized, with values below 0.4 being acceptable, although values below 0.2 are desirable [25].

**Figure 5:**
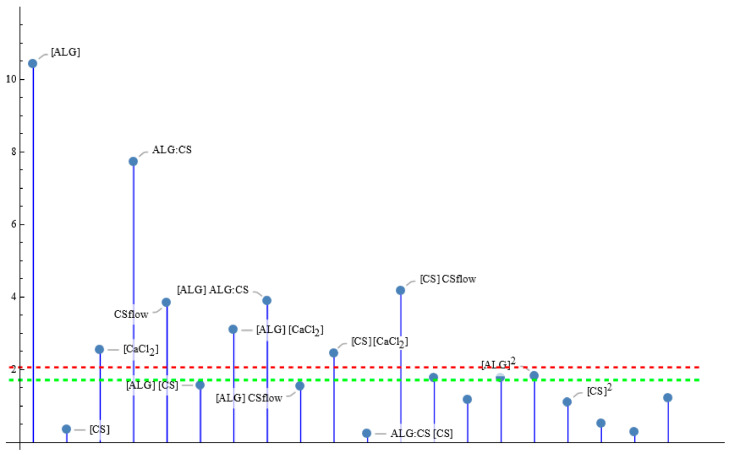
Parameter *t*-test absolute values for the *PDI* fitted model. Values over the green line are significant at 10% significance level, those above the red line are significant for 5% level of significance.

According to the parameter test of significance for the coded variables (Figure 5), four main effects, ‘[ALG]’, ‘[CaCl_2_]’, ‘ALG:CS’ and ‘CSflow’ and four two-factor interactions involving the five factors, are statistically significant for the *PDI* response variable. The usual significance level is 5%; thus, just parameters with a *t*-test significance *p*-value less than 0.05 are initially included in the model, unless there could be other reasonable causes to do so. In this case, the main effect ‘[CS]’ was not significant, but considering its participation in several significant interactions, ‘[CS] [CaCl_2_]’ and ‘[CS] CSflow’, the proposed function for *PDI* includes this main effect as well. Once the model in coded factors is obtained, it is necessary to change to natural variables using the relationship X_i_ = (F_i_ − c_i_)/d_i_, or F_i_ = c_i_ + d_i_ X_i_, where F_i_ denotes the original factor, X_i_ is the corresponding coded one, c_i_ is the central point of the factor and d_i_ is the distance from the center to the extreme levels of the factor (See Appendix C. Relation Between Natural and Codified Factors X_i_). Thus, the final proposal for PDI is expressed in Equation (2).

*PDI* = 0.370 − 0.0104 ALG:CS − 0.0665 CSflow − 0.105 [CaCl_2_] − 0.135 [CS] + 0.446 [ALG]+ 0.197 [CS] CSflow − 0.198 [CS] [CaCl_2_] + 0.252 [ALG] [CaCl_2_] − 0.367 [ALG] ALG:CS (2)

where ‘[ALG]’ refers to ALG concentration, ‘[CS]’ to CS concentration, ‘[CaCl_2_]’ to calcium chloride concentration, ‘ALG:CS’ to ALG:CS volume ratio and ‘CSflow’ to CS flow rate.

The conditions minimizing the *PDI* contain some coordinates equal to (+1) or (−1), so they are in the borders of the cuboidal design region. Thus, the minimum expected value for PDI (0.168) for the experimental region can be reached at {−1, 0.396, 1, 1, −0.515} in coded variables for the five factors, or at [ALG] = 0.30 mg/mL, [CS] = 0.79 mg/mL, [CaCl_2_] = 1.00 mg/mL, ALG:CS volume ratio = 2.50:1.00 mL:mL (molar ratio = 1.2:1.0 mol:mol) and CS flow rate = 0.86 mL/min, in natural variables. Here, the concerns from EE% regarding the ALG:CS ratio should also be taken into account, as all the experiments considered in this work were performed using the protein BSA.

Model for the response variable “*Size*”.

**Figure 6:**
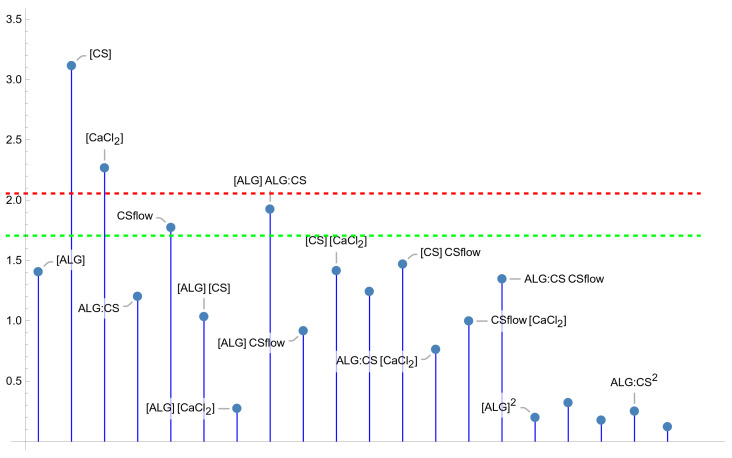
Parameter *t*-test absolute values for the “*size*” fitted model. Values over the green line are significant at 10% significance level, those above the red line are significant for 5% level of significance.

**Figure 7:**
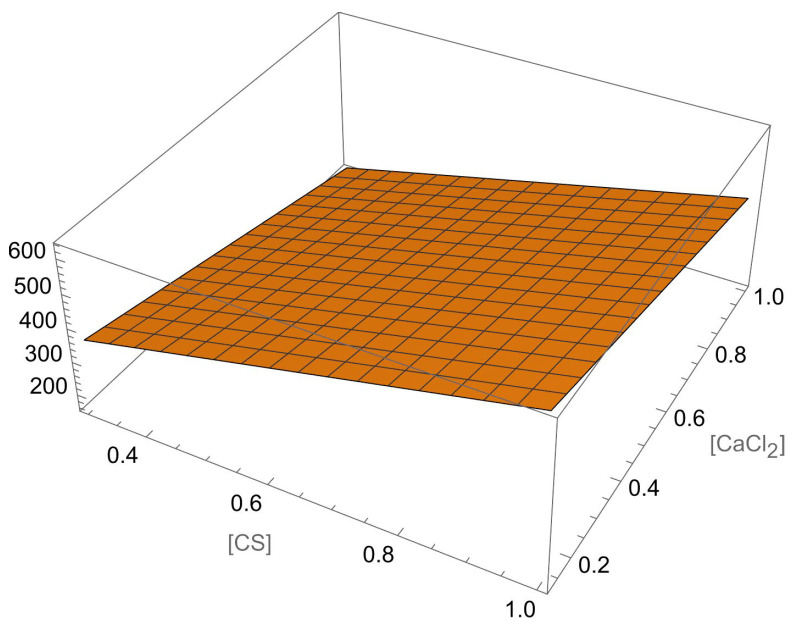
“*Mean size*” as a linear function of [CS] and [CaCl_2_] (5% level of significance).

The modified ionic polyelectrolyte pre-gelation method reported here permits to obtain ALG–CS NPs with a wide range of sizes (144–1965 nm). It should be noted that the optimal range of sizes for drug delivery depends on the administration route and the biological barrier being targeted. However, the preferred typical range of sizes is usually considered to be between 50 and 200 nm for conventional administration routes, i.e., parenteral/oral delivery. Specifically, once in systemic administration, particles with smaller sizes than 20 nm are subject to renal clearance and above 200 nm are removed by the mononuclear phagocyte system (MPS). Both tissue and mucus penetration and cellular uptake are amplified when the particle size is less than 200 nm [26]. Even so, in certain cases, the use of larger NPs may be advisable, as is the case of oral vaccines [27], when a higher payload is required [28], or to increase drug retention in gastrointestinal (inflammatory bowel disease) [29] or intra-articular delivery [30]. Thus, a model to predict the values of this response variable as a function of the significant factors of the synthesis process seems to be very useful. Based on the parameter test of significance (Figure 6), only the main effects ‘[CS]’ and ‘[CaCl_2_]’ are significant at the 5% significance level (the CS flow rate and the interaction of [ALG] with the ALG:CS volume ratio are between 5 and 10%), so, with the usual significance level, this response just depend linearly on the two main factors, as is stated in Equation (3). In Figure 7, the size of the particles is plotted as a function of CaCl_2_ and CS concentrations, showing the linear relation between this response variable and the significant factors, without curvature.

*Size* = 271.629 − 229.797 [CaCl_2_] + 392.503 [CS] (3)

where ‘[CS]’ refers to CS concentration and ‘[CaCl_2_]’ to calcium chloride concentration.

**Figure 8:**
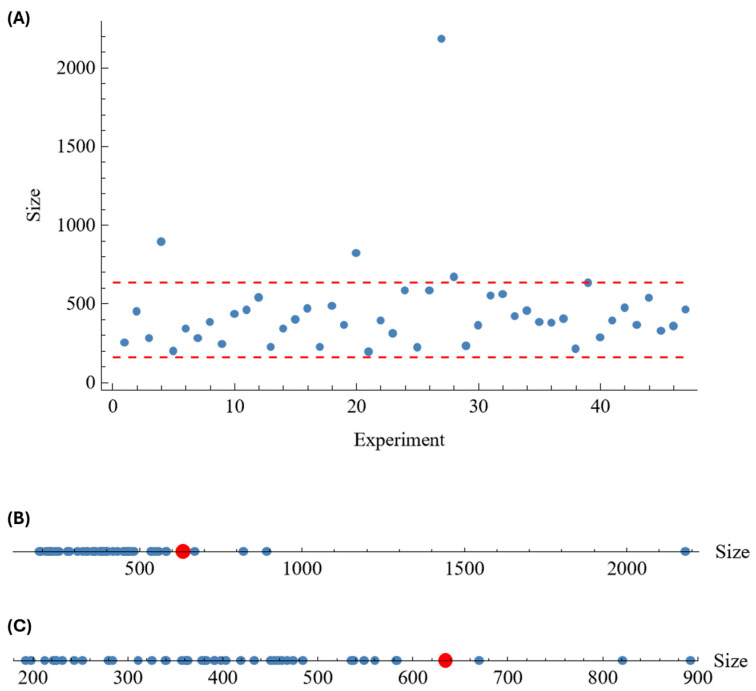
Values of the response “*size*” for the 47 experiments corresponding to the central composite design (Table 5). In (**A**), the red dashed lines show the expected extreme values (160 and 635 nm). In (**B**,**C**), the red dot stands for the expected maximum value (635 nm), excluding in (**C**) the extreme experimental value of experiment #27 (2181 nm).

Using this quite simple model, the minimum expected value of the mean size is close to 160 nm for ‘[CS]’ at the minimum value and ‘[CaCl_2_]’ at the maximum. However, the maximum expected value (635 nm), which can be obtained in the opposite corner (maximum value of ‘[CS]’ and minimum of ‘[CaCl_2_]’), is far from the experimental maximum value. In any case, it should be noted that just 3 out of the 47 experiments have size values significantly greater than 635 nm, 2 of them between 800 and 900 nm and another with an extreme value of 2181 nm (see Figure 8). Furthermore, due to the random component, the experimental “*mean size*” may move beyond the predicted extreme values, but such differences, especially with experiment #27, should come from other causes, and the corresponding values do not represent most of the data (outliers).

Model for the response variable “*Surface charge*” (*ZP*).

Finally, although the ALG–CS NPs generated with the synthesis method reported in this work show, in most cases, highly negative *surface charge* (*ZP*) values (below −30 mV), there are some combinations of factors, which involve an increase of the proportion of CS with respect to ALG, that allow obtaining positive values of ZP (above +20 mV). Therefore, the modelling of this response variable seems to be of great interest for future applications, e.g., interactions with biological membranes.

**Figure 9:**
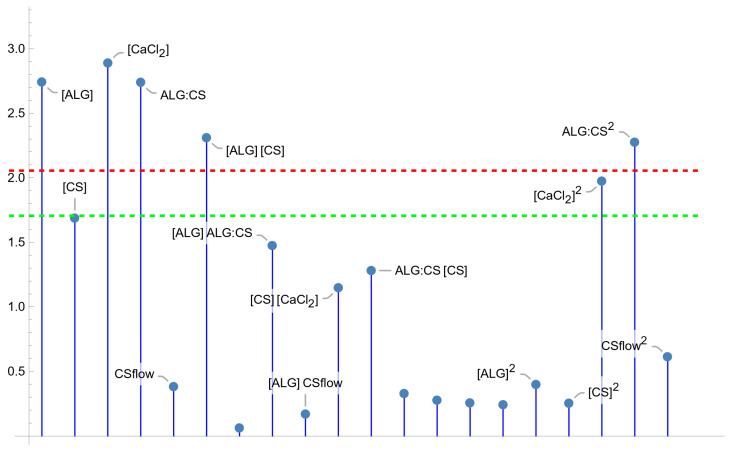
Parameter *t*-test absolute values for the “*ZP*” model. Values over the green line are significant at 10% significance level, those above the red line are significant for 5% level of significance.

Figure 9 shows the parameter test of significance for the coded factors. It can be seen that the model for *ZP* has strong dependence on the ALG:CS volume ratio through the main and the quadratic effect. Something similar can be observed for ‘[CaCl_2_]’, with the main effect at the 5% significance level and the quadratic one very close to this level (*p*-value = 0.059). Furthermore, the main effect ‘[ALG]’ and its interaction with ‘[CS]’ are significant. However, the main effect ‘[CS]’ is just at the border of 10% significance (*p*-value 0.104). Still, due to its participation in the only significant interaction, it seems reasonable to include it in the model. Therefore, the final model can be expressed by Equation (4).

*ZP* = −42.434 + 14.053 [ALG] + 51.103 [CS] + 147.485 [CaCl_2_] − 198.245 ALG:CS − 56.913 [ALG] [CS]−113.315 [CaCl_2_]^2^ + 175.701 ALG:CS^2^ (4)

where ‘[ALG]’ refers to ALG concentration, ‘[CS]’ to CS concentration, ‘[CaCl_2_]’ to calcium chloride concentration and ‘ALG:CS’ to ALG:CS volume ratio.

**Figure 10:**
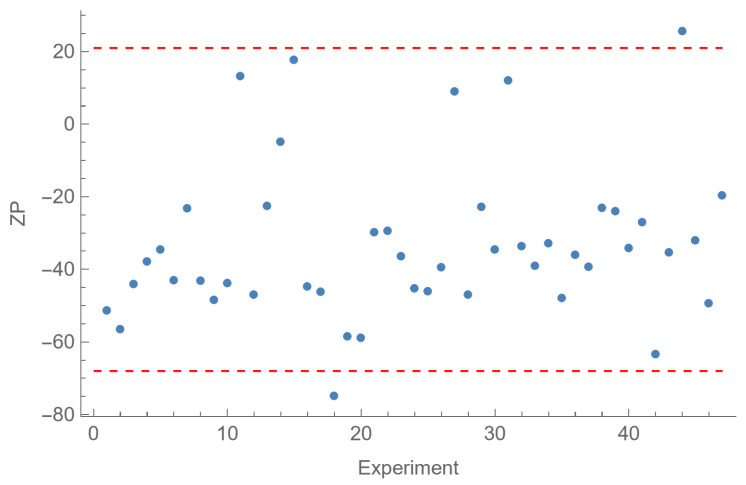
Values of the response “*ZP*” (mV) for the 47 experiments corresponding to the central composite design (CCD) (Table 6 and Table 7). The red dashed lines show the expected extreme values (−67.8 and +21.1 mV).

From the proposed model for *ZP*, the expected minimum value is −67.8 mV for the four coded factor values {0.106, −1, −1, −0.106}, that is [ALG] = 0.69 mg/mL; [CS] = 0.30 mg/mL; [CaCl_2_] = 0.13 mg/mL; ALG:CS volume ratio = 2.50:0.59 mL:mL (molar ratio = 12.4:1.0 mol:mol), in natural variables. The expected maximum is +21.1 mV for the factor values {−1, 1, 0.1, 1}, in coded variables, that is [ALG] = 0.30 mg/mL; [CS] = 1.00 mg/mL; [CaCl_2_] = 0.61 mg/mL; ALG:CS volume ratio = 2.50:1.00 mL:mL (molar ratio = 0.95:1.0 mol:mol), in natural variables. Note that the rest of factors are fixed at the corresponding central levels. In Figure 10, it can be seen that of the 47 samples of the central composite design, just #18, with a value −74.9 mV, is smaller than the expected minimum, and only #44, with a value of 25.6 mV, is greater than the expected maximum, both quite close to the limits. These differences can be explained by the random component that may always appear in the experimentation. Note that, as with *EE*% and *PDI*, the optimal ALG:CS ratios may also vary slightly in this case due to the competition of BSA with ALG for CS reacting groups.

## 4. Conclusions and Future Perspectives

In this work, substantial modifications of the two-step ionic polyelectrolyte pre-gelation method to synthesize alginate–chitosan nanoparticles (ALG-CS NPs) have been reported. The addition of CaCl_2_ to alginate (ALG) to obtain a pregel and the subsequent addition of chitosan (CS) to the pregel to form a polyelectrolyte complex that stabilizes the system were performed in a controlled manner by using syringe pumps and under sonication to enhance the mixing of the phases. This synthesis process was mathematically studied by a two-step design of experiments (DoE)-based methodology. Of the seven factors initially identified as having a possible influence on this technique, only five of them (ALG, CS and CaCl_2_ concentrations, ALG:CS volume ratio and the CS flow rate) showed a significant effect after applying a screening procedure with a 2_IV_^7-3^ fractional design. These five selected factors were subjected to a Response Surface Methodology (RSM) using Central Composite Designs (CCD) to obtain fitted approximated models that correlate these significant factors with the *mean size*, *polydispersity* (*PDI*) and *surface charge* (*ZP*) of the ALG–CS NPs generated and their *encapsulation efficiency* (*EE*%), using bovine serum albumin (BSA) as model drug.

The *mean size* of the ALG–CS NPs generated, whose values were mostly between 200 and 700 nm (although nanoparticles close to 100 nm and larger than 1000 nm were obtained), depends on only two factors (the concentrations of CS and CaCl_2_, i.e., ‘[CS]’ and ‘[CaCl_2_]’) through a plain 3-parameter model. The maximum “*mean size*” expected with the proposed model (635 nm) can be achieved for ‘[CS]’ at the maximum level and ‘[CaCl_2_]’ at the minimum, being located in the opposite corner of the design region the minimum expected value (160 nm) of this response variable. However, it should be noted that the final model proposed for ‘*size*’, although computed taking the statistically significant terms, is quite poor compared to the rest of responses, explaining just 23.6% of the data variability (adjusted R^2^ = 0.201). Trying to improve the model, if the factor effects that are significant at 10% were also added to the model, only a small improvement would be achieved (R^2^ = 0.345, adjusted R^2^ = 0.222). Thus, it is not worth changing the usual assumption (5% of significance) employed in the rest of the analyses. Therefore, the logical explanation is that there might be other non-considered factors that may have influence on this response (and not so much in the other responses). Further research on these influential sources will be the objective of future studies.

Regarding the *surface charge* (“*ZP*”), most of the synthesis conditions yielded highly negative ALG–CS NPs (mainly between −20 and −60 mV), although increasing the mass proportion of CS with respect to ALG also made it possible to obtain positively charged nanoparticles (up to values above +20 mV). This response is dependent on four factors (the concentrations of the reactants and the ALG:CS volume ratio), with its minimum (−67.8 mV) and maximum (+21.1 mV) values obtained for the coded values of the factors ‘[ALG]’, ‘[CS]’, ‘[CaCl_2_]’ and ‘ALG:CS’ of {0.106, −1, −1, −0.106} and {−1, 1, 0.1, 1}, respectively.

Finally, the response variables “*PDI*” and “*EE*%” depend on all the five selected factors after applying the RSM methodology and are susceptible to be optimized (minimized and maximized, respectively). Thus, the minimum “*PDI*” expected value within the experimental region (0.168) can be achieved in the borders of the cuboidal design region, {−1, 0.396, 1, 1, −0.515} in coded variables, and the maximum *EE*% expected (close to the 90%) is obtained at the vertices of the experimental region, {−1, 1, 1, 1, 1} in coded variables.

Table 8 shows a comparative of the values of the four responses for the extreme conditions above, highlighting the extreme values. It can be seen that the conditions maximizing *EE*% are also good for minimizing *PDI*, and conversely (the conditions minimizing *PDI* produce high values of *EE*%). Note that the conditions maximizing *ZP* are also good for both maximizing *EE*% and minimizing *PDI* (last row of Table 8).

In any case, further research needs to be done to propose a reasonable multi-objective function considering at the same time several responses (or even all of them) with weight coefficients depending on the objectives of the researcher. For instance, if the aim is maximizing *EE*% as well as minimizing *PDI*, the function to maximize should be *f* = *w*_1_ *EE*% − *w*_2_ *PDI*, where the weights *w*_1_ and *w*_2_ reflect the overall preferences of the researcher (*w*_1_ = *w*_2_ since maximizing *EE*% seems equally important as minimizing *PDI*). When considering responses of different scales (as in this case), it would be sensible to transform them previously (e.g., getting transformed variables with mean 0 and standard deviation 1) before using them together in the same objective function.

Validation experiments have been performed, especially at or near extreme conditions, proving the goodness of *EE*% and *PDI* models (Table 9). For *ZP* the conditions for the maximum expected value are also confirmed for the experimental values, however, the discrepancy is greater when trying at the conditions related to the minimum expected value, being −46.5 mV the closest value obtained in the environment of these conditions (Table 9). It should be considered that the greater discrepancy between models and actual data will always be at the borders of the design region. Also, note that BSA is included in the CaCl_2_ solution (pH: 6.8) in all cases, which implies that the protein behaves as a negatively charged molecule (pI: 4.7), competing with ALG for CS reacting groups and potentially altering the ALG:CS optimal ratio. It is also worth noting that the proposed models and the optimal conditions of synthesis provided are only valid for specification ranges indicated for reagents and nanoparticle preparation in the “Materials and Methods” section (Section 2.1 and Section 2.2).

In this work, a toolbox of classical designs, including factorial, fractional factorial and non-rotatable central composite designs, has been employed. A complementary approach can be the use of optimal experimental design techniques to obtain the best designs to be employed in the different phases of the study. This is a subject that has experienced high development in recent years, allowing the improvement of any experimental process from different points of view, depending on the objectives of the scientist [31]. Furthermore, in this case, with several responses to be observed, multiresponse models can be considered, studying and developing the correlation structure between them [32,33]. Using this correlation structure, specific designs can be computed for the most-accuracy prediction of the four responses simultaneously, using optimal experimental design approaches. All this can be part of future work.

## Figures and Tables

**Table 1:** Factors and levels considered for the modified ionic polyelectrolyte pregelation method.

Levels	[ALG] (mg/mL)	[CS] (mg/mL)	[CaCl_2_] (mg/mL)	ALG:CS (mL:mL)	ALG:CaCl_2_ (mL:mL)	CaCl_2_ FR (mL/min)	CS FR (mL/min)
−1	0.30	0.30	0.13	2.50:0.25	2.50:0.16	0.50	0.50
+1	1.00	1.00	1.00	2.50:1.00	2.50:0.46	2.00	2.00

ALG: alginate; CS: chitosan; CaCl_2_: calcium chloride; FR: flow rate; −1: low level; +1: high level. Note that ALG:CS molar ratios (mol:mol) are: (−1): 12.7:1.0 and (+1): 3.2:1.0, and ALG:CaCl_2_ molar ratios (mol:mol) are: (−1): 20.2:1.0 and (+1): 3.1:1.0, considering molecular weights of the repeating units of sodium ALG and CS lactate (degree of deacetylation ≤ 95%).

**Table 2:** Values of *size* and *PDI* for several conditions, using sonication or magnetic stirring.

	Experimental Conditions							Responses by Mixing Technique			
								Sonication		Mag.stir.	
	[ALG] (mg/mL)	[CS] (mg/mL)	[CaCl_2_] (mg/mL)	ALG:CS (mL:mL)	ALG:CaCl_2_ (mL:mL)	FR (mL/min) CaCl_2_ CS		*Size* (nm)	*PDI* (0–1)	*Size* (nm)	*PDI* (0–1)
1	0.30	1.00	1.00	2.50:0.25	2.50:0.16	0.50	2.00	281.1	0.298	525.0	0.383
2	0.30	0.30	1.00	2.50:0.25	2.50:0.46	2.00	2.00	278.6	0.258	520.9	0.352
3	1.00	1.00	0.13	2.50:1.00	2.50:0.16	0.50	0.50	524.0	0.482	1554.0	0.704
4	1.00	1.00	1.00	2.50:1.00	2.50:0.46	2.00	2.00	572.1	0.550	1652.0	0.834
5	0.30	1.00	1.00	2.50:1.00	2.50:0.16	2.00	0.50	656.1	0.297	1629.0	0.924

ALG: sodium alginate; CS: chitosan lactate; CaCl_2_: calcium chloride; FR: flow rates; Mag.stir.: magnetic stirring; PDI: polydispersity index.

**Table 3:** Values of response variables (*size*, *PDI*, *ZP*, *EE*%) as a function of the levels of the factors.

	Factors and Their Values							Responses			
	[ALG] (mg/mL)	[CS] (mg/mL)	[CaCl_2_] (mg/mL)	ALG:CS (mL:mL)	ALG:CaCl_2_ (mL:mL)	FR (mL/min) CaCl_2_ CS		*Size* (nm)	*PDI* (0–1)	*ZP* (mV)	*EE*% (%)
1	0.30	0.30	0.13	2.50:1.00	2.50:0.16	2.00	2.00	191.6	0.279	−38.5	77.1
2	0.30	0.30	0.13	2.50:1.00	2.50:0.46	2.00	2.00	144.0	0.262	−31.4	60.4
3	0.30	0.30	0.13	2.50:0.25	2.50:0.16	2.00	2.00	183.2	0.303	−46.4	72.1
4	0.30	0.30	0.13	2.50:0.25	2.50:0.46	2.00	2.00	146.2	0.293	−44.1	42.2
5	0.30	0.30	1.00	2.50:1.00	2.50:0.16	2.00	2.00	267.9	0.265	−25.6	89.4
6	0.30	0.30	1.00	2.50:1.00	2.50:0.46	2.00	2.00	332.9	0.204	−19.6	62.0
7	0.30	0.30	1.00	2.50:0.25	2.50:0.16	2.00	2.00	276.7	0.306	−33.6	76.4
8	0.30	0.30	1.00	2.50:0.25	2.50:0.46	2.00	2.00	292.9	0.243	−29.2	72.8
9	0.30	1.00	0.13	2.50:1.00	2.50:0.16	2.00	2.00	1965.0	0.563	14.0	99.6
10	0.30	1.00	0.13	2.50:1.00	2.50:0.46	2.00	2.00	1026.0	0.381	17.0	99.3
11	0.30	1.00	0.13	2.50:0.25	2.50:0.16	2.00	2.00	240.8	0.371	−39.5	77.0
12	0.30	1.00	0.13	2.50:0.25	2.50:0.46	2.00	2.00	169.9	0.322	−43.1	62.5
13	0.30	1.00	1.00	2.50:1.00	2.50:0.16	2.00	2.00	627.9	0.345	18.2	99.1
14	0.30	1.00	1.00	2.50:1.00	2.50:0.46	2.00	2.00	595.6	0.229	32.0	98.8
15	0.30	1.00	1.00	2.50:0.25	2.50:0.16	2.00	2.00	261.2	0.389	−37.6	83.3
16	0.30	1.00	1.00	2.50:0.25	2.50:0.46	2.00	2.00	403.9	0.227	−24.5	81.1
17	1.00	0.30	0.13	2.50:1.00	2.50:0.16	2.00	2.00	724.7	0.584	−78.1	27.4
18	1.00	0.30	0.13	2.50:1.00	2.50:0.46	2.00	2.00	485.4	0.609	−55.9	0.1
19	1.00	0.30	0.13	2.50:0.25	2.50:0.16	2.00	2.00	481.4	0.787	−69.4	23.2
20	1.00	0.30	0.13	2.50:0.25	2.50:0.46	2.00	2.00	361.8	0.969	−54.6	5.0
21	1.00	0.30	1.00	2.50:1.00	2.50:0.16	2.00	2.00	404.1	0.589	−68.1	14.6
22	1.00	0.30	1.00	2.50:1.00	2.50:0.46	2.00	2.00	339.1	0.587	−56.0	19.4
23	1.00	0.30	1.00	2.50:0.25	2.50:0.16	2.00	2.00	342.8	0.852	−65.4	39.3
24	1.00	0.30	1.00	2.50:0.25	2.50:0.46	2.00	2.00	555.6	0.606	−45.1	34.6
25	1.00	1.00	0.13	2.50:1.00	2.50:0.16	2.00	2.00	704.4	0.539	−59.0	41.5
26	1.00	1.00	0.13	2.50:1.00	2.50:0.46	2.00	2.00	663.9	0.540	−61.9	24.6
27	1.00	1.00	0.13	2.50:0.25	2.50:0.16	2.00	2.00	954.3	0.913	−64.9	29.3
28	1.00	1.00	0.13	2.50:0.25	2.50:0.46	2.00	2.00	804.5	0.928	−63.8	12.1
29	1.00	1.00	1.00	2.50:1.00	2.50:0.16	2.00	2.00	559.9	0.579	−55.1	44.1
30	1.00	1.00	1.00	2.50:1.00	2.50:0.46	2.00	2.00	511.2	0.438	−35.2	89.0
31	1.00	1.00	1.00	2.50:0.25	2.50:0.16	2.00	2.00	477.6	0.943	−61.9	31.0
32	1.00	1.00	1.00	2.50:0.25	2.50:0.46	2.00	2.00	453.7	1.000	−49.1	25.0

ALG: sodium alginate; CS: chitosan lactate; CaCl_2_: calcium chloride; FR: flow rates; PDI: polydispersity index; ZP: Z Potential (surface charge); EE%: encapsulation efficiency.

**Table 4:** Design matrix for the 2_IV_^7-3^ fractional design (screening procedure), including factors, levels and response values.

	Factors and Their Values							Response Variables			
	x[1]	x[2]	x[3]	x[4]	x[5]	x[6]	x[7]	*Size* (nm)	*PDI*	*ZP* (mV)	*EE*% (%)
y[1]	−1	−1	−1	−1	−1	−1	−1	104.7	0.484	−52.4	20.3
y[2]	+1	−1	−1	−1	+1	−1	+1	434.0	0.839	−52.1	6.0
y[3]	−1	+1	−1	−1	+1	+1	−1	152.1	0.525	−50.2	25.0
y[4]	+1	+1	−1	−1	−1	+1	+1	901.3	1.000	−63.7	21.5
y[5]	−1	−1	+1	−1	+1	+1	+1	278.6	0.258	−23.0	69.3
y[6]	+1	−1	+1	−1	−1	+1	−1	296.9	0.917	−47.4	26.2
y[7]	−1	+1	+1	−1	−1	−1	+1	281.1	0.298	−32.5	78.0
y[8]	+1	+1	+1	−1	+1	−1	−1	353.9	0.582	−46.5	24.6
y[9]	−1	−1	−1	+1	−1	+1	+1	168.6	0.326	−42.1	81.4
y[10]	+1	−1	−1	+1	+1	+1	−1	354.7	0.545	−66.4	16.4
y[11]	−1	+1	−1	+1	+1	−1	+1	1886.0	0.687	8.7	99.2
y[12]	+1	+1	−1	+1	−1	−1	−1	524.0	0.482	−52.8	47.2
y[13]	−1	−1	+1	+1	+1	−1	−1	347.3	0.211	−22.1	85.0
y[14]	+1	−1	+1	+1	−1	−1	+1	393.2	0.617	−53.5	22.9
y[15]	−1	+1	+1	+1	−1	+1	−1	656.1	0.297	19.5	99.4
y[16]	+1	+1	+1	+1	+1	+1	+1	572.1	0.550	−41.9	82.6

x[1]: ALG concentration (mg/mL); x[2]: CS concentration (mg/mL); x[3]: CaCl_2_ concentration (mg/mL); x[4]: ALG:CS volume ratio (mL:mL); x[5]: ALG:CaCl_2_ volume ratio (mL:mL); x[6]: CaCl_2_ flow rate; x[7]: CS flow rate; PDI: polydispersity index; ZP: Z Potential; EE%: encapsulation efficiency; y[1]–y[16]: number of runs.

**Table 5:** Factors and levels used in the RSM procedure for the natural and coded variables.

Levels	[ALG] (mg/mL)	[CS] (mg/mL)	[CaCl_2_] (mg/mL)	ALG:CS (mL:mL)	ALG:CaCl_2_ (mL:mL)	CaCl_2_ FR (mL/min)	CS FR (mL/min)
−1	0.30	0.30	0.13	2.50:0.25	2.50:0.31	1.25	0.50
0	0.65	0.65	0.57	2.50:0.63			1.25
+1	1.00	1.00	1.00	2.50:1.00			2.00

ALG: alginate; CS: chitosan; CaCl_2_: calcium chloride; FR: flow rate; +1: high level; 0: center level; −1: low level. Note that ALG:CS molar ratios (mol:mol) are: (−1): 12.7:1.0; (0): 5.1:1.0; (+1): 3.2:1.0; and ALG:CaCl_2_ molar ratio (mol:mol) for the central level (0) is: 5.2:1.0, considering molecular weights of the repeating units of sodium ALG and CS lactate (degree of deacetylation ≤ 95%).

**Table 6:** CCD and experimental values of responses for RSM procedure (2^5^ factorial design).

	Factors					Response Variables			
	x[1]	x[2]	x[3]	x[4]	x[7]	Size (nm)	*PDI*	*ZP* (mV)	*EE*% (%)
y[1]	−1	−1	−1	−1	−1	252.4	0.480	−51.4	17.5
y[2]	+1	−1	−1	−1	−1	451.0	0.727	−56.6	6.3
y[3]	−1	+1	−1	−1	−1	280.1	0.498	−44.1	22.8
y[4]	+1	+1	−1	−1	−1	892.8	0.617	−38.0	18.1
y[5]	−1	−1	+1	−1	−1	198.3	0.508	−34.7	12.6
y[6]	+1	−1	+1	−1	−1	340.8	0.815	−43.1	24.7
y[7]	−1	+1	+1	−1	−1	280.2	0.308	−23.3	33.8
y[8]	+1	+1	+1	−1	−1	381.8	0.640	−43.2	18.6
y[9]	−1	−1	−1	+1	−1	243.6	0.349	−48.5	3.8
y[10]	+1	−1	−1	+1	−1	433.7	0.483	−43.9	14.9
y[11]	−1	+1	−1	+1	−1	458.5	0.271	13.2	99.8
y[12]	+1	+1	−1	+1	−1	537.8	0.405	−47.1	37.5
y[13]	−1	−1	+1	+1	−1	224.1	0.269	−22.6	83.6
y[14]	+1	−1	+1	+1	−1	339.5	0.493	−4.9	19.4
y[15]	−1	+1	+1	+1	−1	398.5	0.167	17.7	99.9
y[16]	+1	+1	+1	+1	−1	467.9	0.356	−44.8	42.6
y[17]	−1	−1	−1	−1	+1	225.1	0.382	−46.3	41.4
y[18]	+1	−1	−1	−1	+1	484.5	0.779	−74.9	2.3
y[19]	−1	+1	−1	−1	+1	363.7	0.503	−58.5	60.2
y[20]	+1	+1	−1	−1	+1	821.0	0.890	−58.9	11.8
y[21]	−1	−1	+1	−1	+1	192.8	0.333	−29.9	70.2
y[22]	+1	−1	+1	−1	+1	391.5	0.814	−29.5	43.7
y[23]	−1	+1	+1	−1	+1	311.2	0.406	−36.5	79.4
y[24]	+1	+1	+1	−1	+1	583.9	0.897	−45.4	44.1
y[25]	−1	−1	−1	+1	+1	221.8	0.351	−46.2	64.3
y[26]	+1	−1	−1	+1	+1	583.0	0.550	−39.5	5.6
y[27]	−1	+1	−1	+1	+1	2181.0	0.803	8.9	99.8
y[28]	+1	+1	−1	+1	+1	670.0	0.564	−47.1	26.1
y[29]	−1	−1	+1	+1	+1	231.3	0.267	−22.9	61.9
y[30]	+1	−1	+1	+1	+1	361.0	0.580	−34.6	33.9
y[31]	−1	+1	+1	+1	+1	549.3	0.261	12.0	99.7
y[32]	+1	+1	+1	+1	+1	560.5	0.528	−33.7	34.9

x[1]: ALG concentration (mg/mL); x[2]: CS concentration (mg/mL); x[3]: CaCl_2_ concentration (mg/mL); x[4]: ALG:CS volume ratio; x[7] CS flow rate; y[1]–y[32]: number of runs; −1: low level; +1: high level. Note that in this design, the experiments were carried out using the center level (0) for the factors x[5]: ALG:CaCl_2_ volume ratio and x[6]: CaCl_2_ flow rate.

**Table 7:** CCD and experimental values of responses for RSM procedure (central and axial points).

	Factors					Response Variables			
	x[1]	x[2]	x[3]	x[4]	x[7]	*Size* (nm)	*PDI*	*ZP* (mV)	*EE*% (%)
y[33]	0	0	0	0	0	419.1	0.388	−39.1	25.7
y[34]	0	0	0	0	0	454.8	0.390	−32.9	26.4
y[35]	0	0	0	0	0	383.1	0.535	−48.0	17.1
y[36]	0	0	0	0	0	378.5	0.458	−36.1	15.1
y[37]	0	0	0	0	0	403.3	0.496	−39.4	20.0
y[38]	−1	0	0	0	0	212.8	0.226	−23.2	37.1
y[39]	+1	0	0	0	0	632.0	0.521	−24.1	14.7
y[40]	0	−1	0	0	0	284.4	0.539	−34.3	6.2
y[41]	0	+1	0	0	0	391.4	0.468	−27.1	20.3
y[42]	0	0	−1	0	0	474.4	0.493	−63.4	11.5
y[43]	0	0	+1	0	0	363.1	0.461	−35.4	36.9
y[44]	0	0	0	−1	0	535.6	0.602	25.6	0.1
y[45]	0	0	0	+1	0	325.9	0.331	−32.1	22.0
y[46]	0	0	0	0	−1	356.9	0.481	−49.5	24.0
y[47]	0	0	0	0	+1	462.3	0.536	−19.7	13.2

x[1]: ALG concentration (mg/mL); x[2]: CS concentration (mg/mL); x[3]: CaCl_2_ concentration (mg/mL); x[4]: ALG:CS volume ratio; x[7] CS flow rate; y[33]–y[47]: number of runs; −1: low level; +1: high level; 0: center level. Note that in this design, the experiments were carried out using the center level (0) for the factors x[5]: ALG:CaCl_2_ volume ratio and x[6]: CaCl_2_ flow rate.

**Table 8:** Comparative of the expected values of the four responses at the factor conditions for the extreme values of each one. Expected extreme values are shown in boldface style.

Extremes	Coded Factors {X1, X2, X3, X4, X7}	*EE* (%)	*PDI*	*Size* (nm)	*ZP* (mV)
Max. *EE*%	{−1, 1, 1, 1, 1}	**86.7**	0.282	434.0	+7.4
Min. *PDI*	{−1, 0.396, 1, 1, −0.515}	64.4	**0.168**	351.0	+0.2
Min. *Size*	{0, −1, 1, 0, 0}	15.2	0.454	**160.0**	−50.1
Max. *Size*	{0, 1, −1, 0, 0}	17.6	0.515	**634.0**	−57.2
Min. *ZP*	{0.106, −1, −1, −0.106, 0}	0.0	0.474	360.0	**−67.8**
Max. *ZP*	{−1, 1, 0.1, 1, 0}	72.3	0.273	524.0	**+21.1**

X1, X2 and X3: ALG, CS and CaCl_2_ concentrations (mg/mL); X4: ALG:CS volume ratio (mL:mL); X7: CS flow rate.

**Table 9:** Validation runs at the extreme conditions of the responses.

Predicted Extreme Conditions {X1, X2, X3, X4, X5, X6, X7} (Coded Factors)		Observed (*n* = 3)			Predicted		
		*PDI* (0–1)	*ZP* (mV)	*EE*% (%)	*PDI* (0–1)	*ZP* (mV)	*EE*% (%)
*EE*% (optimum)	{−1, 1, 1, 1, 0, 0, 1}			82.2 ± 5.7			86.7
*PDI* (optimum)	{−1, 0.396, 1, 1, 0, 0, −0.515}	0.203 ± 0.07			0.168		
*ZP* (at max.)	{−1, 1, 0.1, 1, 0, 0, 0}		+23.3 ± 2.7			+21.1	
*ZP* (at min.)	{0.106, −1, −1, −0.106, 0, 0, 0}		−34.7 ± 2.0			−67.8	
*ZP* (near min.)	{1, 1, 1, −0.162, 0, 0, 0}		−46.5 ± 1.9			−55.94	

X1, X2 and X3: ALG, CS and CaCl_2_ concentrations (mg/mL); X4: ALG:CS volume ratio (mL:mL); X5: ALG:CaCl_2_ volume ratio (mL:mL); X6: CaCl_2_ flow rate; X7 CS flow rate. Note that the “size” model was not validated since it explains just 23.6% of the data variability.

**Figure A1:**
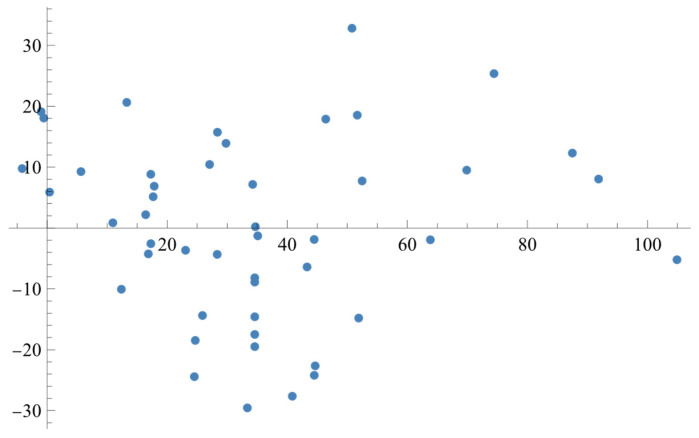
Residuals vs predicted values for *EE*%.

**Figure A2:**
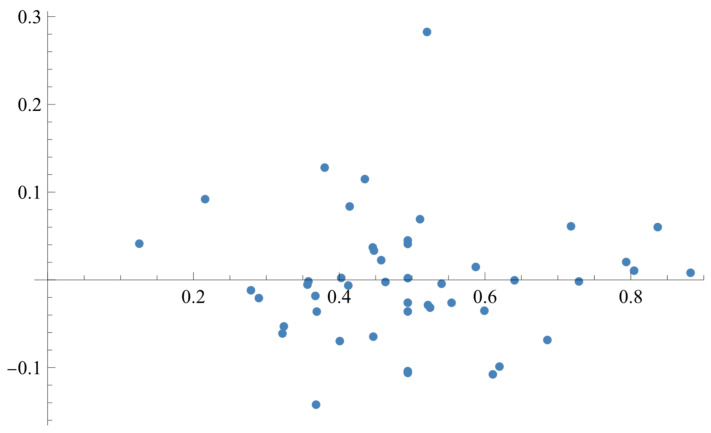
Residuals vs predicted values for *PDI*.

**Figure A3:**
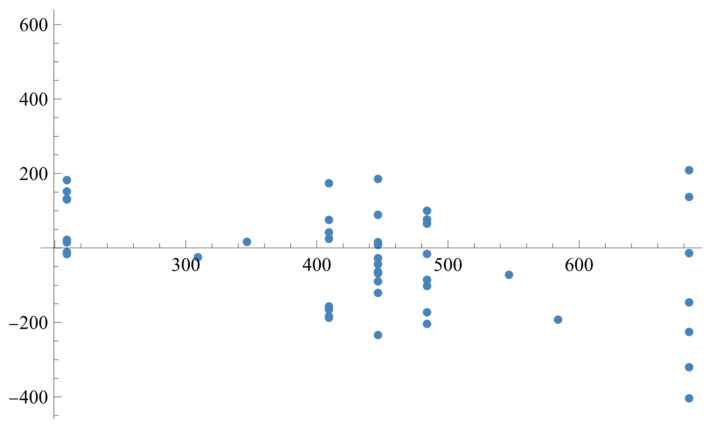
Residuals vs predicted values for *Size*.

**Figure A4:**
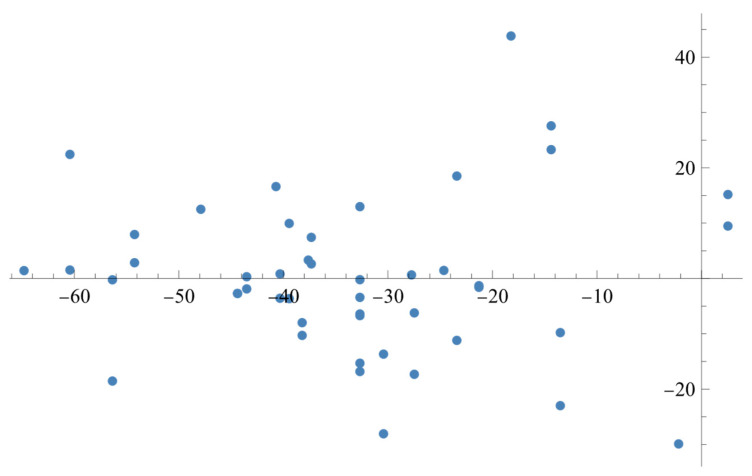
Residuals vs predicted values for *ZP*.

## Data Availability

The original contributions presented in this study are included in the article. Further inquiries can be directed to the corresponding authors.

## Appendix A. Detailed Results of the Screening Procedure

Results performed with SPSS v.22 software for the codified variables X1, …, X7, coming, respectively, from [ALG], [CS], [CaCl_2_], ALG:CS volume ratio, ALG:CaCl_2_ volume ratio, CaCl_2_ flow rate and CS flow rate. The variables significant at 5% are highlighted in green, and the ones significant at 10% are highlighted in blue.

***Size***					
**Source**	**Sum of Squares**	**DF**	**Mean Square**	**F Ratio**	**Significance (*p* Value)**
X1	8632.181	1	8632.181	0.092	0.763
X2	783899.592	1	783899.592	8.365	0.006
X3	360668.661	1	360668.661	3.849	0.057
X4	634254.255	1	634254.255	6.768	0.013
X5	6683.011	1	6683.011	0.071	0.791
X6	191329.357	1	191329.357	2.042	0.161
X7	327591.486	1	327591.486	3.496	0.069
Operator	239938.965	1	239938.965	2.560	0.117
Error	3748499.534	40	93712.488		
Total	6539035.142	48			
R square = 0.427 (Adjusted R square = 0.312). DF: degree of freedom.					

***PDI***					
**Source**	**Sum of Squares**	**DF**	**Mean Square**	**F Ratio**	**Significance (*p* Value)**
X1	0.851	1	0.851	48.703	0.000
X2	0.008	1	0.008	0.470	0.497
X3	0.075	1	0.075	4.288	0.045
X4	0.300	1	0.300	17.157	0.000
X5	0.016	1	0.016	0.929	0.341
X6	0.005	1	0.005	0.294	0.590
X7	0.007	1	0.007	0.400	0.531
Operator	0.083	1	0.083	4.721	0.036
Error	0.699	40	0.017		
Total	3.024	48			
R square = 0.769 (Adjusted R square = 0.723). DF: degree of freedom.					

***ZP***					
**Source**	**Sum of Squares**	**DF**	**Mean Square**	**F Ratio**	**Significance (*p* Value)**
X1	14368.610	1	14368.610	72.386	0.000
X2	1240.597	1	1240.597	6.250	0.017
X3	1636.696	1	1636.696	8.245	0.007
X4	3310.687	1	3310,687	16.678	0.000
X5	600.818	1	600.818	3.027	0.090
X6	131.035	1	131.035	0.660	0.421
X7	25.377	1	25.377	0.128	0.723
Operator	1216.863	1	1216.863	6.130	0.018
Error	7940.023	40	198.501		
Total	31947.701	48			
R square= 0.751 (Adjusted R square = 0.702). DF: degree of freedom.					

***EE*%**					
**Source**	**Sum of Squares**	**DF**	**Mean Square**	**F Ratio**	**Significance (*p* Value)**
X1	16534.109	1	16534.109	71.552	0.000
X2	3595.261	1	3595.261	15.559	0.000
X3	2948.840	1	2948.840	12.761	0.001
X4	4227.427	1	4227.427	18.294	0.000
X5	349.621	1	349.621	1.513	0.226
X6	11.258	1	11.258	0.049	0.826
X7	636.110	1	636.110	2.753	0.105
Operator	34.793	1	34.793	0.151	0.700
Error	9243.190	40	231.080		
Total	47221.977	48			
R square= 0.804 (Adjusted R square = 0.765). DF: degree of freedom.					

From the results, it seems that X1, X2, X3, X4 are clearly important for most of the response variables. Conversely, X6 was not significant for any of the responses. X5 could be considered just for the response *ZP*, and even that with significance 0.09, thus it can be discarded. Finally, X7 appears in two cases with significance between 5% and 10% but, considering that the preliminary experiments were performed for a constant value of X7 (and, thus, they did not provide information about this factor’s influence), it seems sensible to keep it in the model, just in case. The operator’s influence was not significant for the analysis performed using just the 16 experiments of the 2_IV_^7-3^ fractional design, but when also considering the preliminary experiments, it appears as influential in two of the responses, reducing the non-explained variability. Thus, it is possible to talk about a ‘training effect’, in which there may have been some influence of the operator in the first experiments, those done to set the levels of the factors, but that influence is no longer observed later in the experiments carried out to adjust the models.

* * 

**Confounding structure for the 2_IV_^7-3^ fractional design (Screening Procedure)**

*Generating equations:*

E = ABC, F = BCD, G = ACD

Defining relations:

I = ABCE = BCDF = ADEF = ACDG = BDEG = CEFG = ABFG

Two-factor interactions:

AB = CE = FG

AC = BE = DG

AD = EF = CG

BC = AE = DF

BD = CF = EG

CD = BF = AG

AF = DE = BG

## Appendix B. Model Statistics for RSM Models

*EE*%

**Table A1:** ANOVA and Estimated Parameters Table for the response variable *EE*%.

Term	DF	SS	MS	F-Statistic	*p*-Value (ANOVA)	Estimate	Standard Error	t-Statistic	*p*-Value (Coeff)
1						16.3702	3.83514	4.26848	2.31450 × 10^−04^
X1	1	10,189.7	10,189.7	61.1947	2.68580 × 10^−08^	−17.3118	2.21301	−7.82271	2.68580 × 10^−08^
X2	1	3342.25	3342.25	20.072	1.32551 × 10^−04^	9.91471	2.21301	4.4808	1.32551 × 10^−04^
X3	1	2580.42	2580.42	15.4969	5.51437 × 10^−04^	8.71176	2.21301	3.9366	5.51437 × 10^−04^
X4	1	3442.13	3442.13	20.6719	1.11238 × 10^−04^	10.00618	2.21301	4.54663	1.11238 × 10^−04^
X7	1	1329.38	1329.38	7.98362	8.95019 × 10^−03^	6.25294	2.21301	2.82553	8.95019 × 10^−03^
X1 X2	1	772.245	772.245	4.63775	4.07254 × 10^−02^	−4.9125	2.28112	−2.15354	4.07254 × 10^−02^
X1 X3	1	1.90125	1.90125	0.011418	9.15724 × 10^−01^	0.24375	2.28112	0.106855	9.15724 × 10^−01^
X1 X4	1	1647.38	1647.38	8.89342	4.12362 × 10^−03^	−7.7175	2.28112	−3.14538	4.12362 × 10^−03^
X1 X7	1	1044.25	1044.25	6.27126	1.88751 × 10^−02^	−5.7125	2.28112	−2.50425	1.88751 × 10^−02^
X2 X3	1	427.781	427.781	2.56906	1.21053 × 10^−01^	−3.65625	2.28112	−1.60283	1.21053 × 10^−01^
X2 X4	1	1044.24	1044.24	6.27126	1.88751 × 10^−02^	5.7125	2.28112	2.50425	1.88751 × 10^−02^
X2 X7	1	103.68	103.68	0.622655	4.37199 × 10^−01^	−1.8	2.28112	−0.789085	4.37199 × 10^−01^
X3 X4	1	15.9613	15.9613	0.095856	7.59328 × 10^−01^	−0.70625	2.28112	−0.309606	7.59328 × 10^−01^
X3 X7	1	54.6013	54.6013	0.32791	5.71811 × 10^−01^	1.30625	2.28112	0.572635	5.71811 × 10^−01^
X4 X7	1	946.125	946.125	5.682	2.47296 × 10^−02^	−5.4375	2.28112	−2.38369	2.47296 × 10^−02^
X1^2^	1	4738.91	4738.91	28.4597	1.39201 × 10^−05^	12.3359	8.21332	1.50194	1.45162 × 10^−01^
X2^2^	1	107.423	107.423	0.645133	4.29140 × 10^−01^	−0.314122	8.21332	−0.0382455	9.69784 × 10^−01^
X3^2^	1	374.878	374.878	2.25135	1.45545 × 10^−01^	10.6359	8.21332	1.29496	2.06717 × 10^−01^
X4^2^	1	4.653	4.653	0.0279438	8.68534 × 10^−01^	−2.51412	8.21332	−0.306103	7.61964 × 10^−01^
X7^2^	1	62.5981	62.5981	0.375936	5.45113 × 10^−01^	5.03588	8.21332	0.613136	5.45113 × 10^−01^
Error	26	4329.33	166.513						
Total	46	36,559.8							

DF = Degrees of Freedom; SS = Sum of Squares; MS = Mean Square. *p*-values shown in scientific notation (E). Intercept is labeled ‘1’. Blank cells indicate that the metric is not applicable to that term. R^2^ = 0.882, Adjusted R^2^ = 0.790.

For the final model: R^2^ = 0.720, Adjusted R^2^ = 0.643.

Standard error prediction at the optimal conditions (in fact at any vertex): 9.600.

*PDI*

**Table A2:** ANOVA and Estimated Parameters Table for the response variable *PDI*.

Term	DF	SS	MS	F-Statistic	*p*-Value (ANOVA)	Estimate	Standard Error	t-Statistic	*p*-Value (Coeff)
1						0.454267	0.0209365	21.6974	3.51266 × 10^−18^
X1	1	0.538021	0.538021	108.42	9.07679 × 10^−11^	0.125794	0.0120811	10.4125	9.07679 × 10^−11^
X2	1	5.52029 × 10^−04^	5.52029 × 10^−04^	0.111243	7.41409 × 10^−01^	−0.00402941	0.0120811	−0.333531	7.41409 × 10^−01^
X3	1	0.0319342	0.0319342	6.43526	1.75309 × 10^−02^	−0.0306471	0.0120811	−2.53678	1.75309 × 10^−02^
X4	1	0.295742	0.295742	59.5968	3.42816 × 10^−08^	−0.0932647	0.0120811	−7.7199	3.42816 × 10^−08^
X7	1	0.073145	0.073145	14.7399	7.09992 × 10^−04^	0.0463824	0.0120811	3.83926	7.09992 × 10^−04^
X1 X2	1	0.0120901	0.0120901	2.43635	1.30642 × 10^−01^	−0.0194375	0.0124529	−1.56088	1.30642 × 10^−01^
X1 X3	1	0.0469711	0.0469711	9.46543	4.88289 × 10^−03^	0.0383125	0.0124529	3.07659	4.88289 × 10^−03^
X1 X4	1	0.0741125	0.0741125	14.9349	6.64911 × 10^−04^	−0.048125	0.0124529	−3.86456	6.64911 × 10^−04^
X1 X7	1	0.0116281	0.0116281	2.34325	1.37904 × 10^−01^	0.0190625	0.0124529	1.53077	1.37904 × 10^−01^
X2 X3	1	0.0291611	0.0291611	5.87643	2.26016 × 10^−02^	−0.0301875	0.0124529	−2.42414	2.26016 × 10^−02^
X2 X4	1	0.002645	0.002645	0.053301	8.19223 × 10^−01^	0.002875	0.0124529	0.23087	8.19223 × 10^−01^
X2 X7	1	0.0859051	0.0859051	17.3113	3.07136 × 10^−04^	0.0518125	0.0124529	4.16068	3.07136 × 10^−04^
X3 X4	1	0.0153125	0.0153125	3.08571	9.07560 × 10^−02^	−0.021875	0.0124529	−1.75662	9.07560 × 10^−02^
X3 X7	1	0.00667013	0.00667013	1.34414	2.56843 × 10^−01^	−0.0144375	0.0124529	−1.15937	2.56843 × 10^−01^
X4 X7	1	0.0153125	0.0153125	3.08571	9.07560 × 10^−02^	0.021875	0.0124529	1.75662	9.07560 × 10^−02^
X1^2^	1	0.00634511	0.00634511	1.27864	2.68475 × 10^−01^	−0.0813092	0.0448374	−1.81342	8.13265 × 10^−02^
X2^2^	1	0.027223	0.027223	5.45877	2.71030 × 10^−02^	0.0486908	0.0448374	1.08594	2.87470 × 10^−01^
X3^2^	1	0.00512686	0.00512686	1.03314	3.18787 × 10^−01^	0.0022902	0.0448374	0.049919	6.24814 × 10^−01^
X4^2^	1	0.00153762	0.00153762	0.309854	5.82530 × 10^−01^	0.0116908	0.0448374	0.260739	7.96348 × 10^−01^
X7^2^	1	0.00711559	0.00711559	1.43391	2.41932 × 10^−01^	0.0536908	0.0448374	1.19746	2.41932 × 10^−01^
Error	26	0.129022	0.00496238						
Total	46	1.41319							

DF = Degrees of Freedom; SS = Sum of Squares; MS = Mean Square. *p*-values shown in scientific notation (E). Intercept is labeled ‘1’. Blank cells indicate not applicable. R^2^ = 0.909, Adjusted R^2^ = 0.838.

For the final model: R^2^ = 0.831, Adjusted R^2^ = 0.796.

Standard error prediction at the optimal conditions {−1, 0.396, 1, 1, −0.515} of {X1, X2, X3, X4, X7}: 0.0341.

*Size*

**Table A3:** ANOVA and Estimated Parameters Table for the response variable *Size*.

Term	DF	SS	MS	F-Statistic	*p*-Value (ANOVA)	Estimate	Standard Error	t-Statistic	*p*-Value (Coeff)
1						396.92	76.4164	5.19417	2.01450 × 10^−05^
X1	1	130,696.	130,696.	1.97699	1.71547 × 10^−01^	62.	44.095	1.40605	1.71547 × 10^−01^
X2	1	641,658.	641,658.	9.70612	4.43877 × 10^−03^	137.376	44.095	3.11547	4.43877 × 10^−03^
X3	1	339,740.	339,740.	5.13912	3.19415 × 10^−02^	−99.9616	44.095	−2.26696	3.19415 × 10^−02^
X4	1	95,368.2	95,368.2	1.44236	2.40547 × 10^−01^	52.9618	44.095	1.20108	2.40547 × 10^−01^
X7	1	207,480.	207,480.	3.13848	8.81871 × 10^−02^	78.1176	44.095	1.77158	8.81871 × 10^−02^
X1 X2	1	70,537.7	70,537.7	1.067	3.11138 × 10^−01^	−46.95	45.4521	−1.03296	3.11138 × 10^−01^
X1 X3	1	4841.28	4841.28	0.0732323	8.07882 × 10^−01^	12.3	45.4521	0.270615	8.07882 × 10^−01^
X1 X4	1	244,685.	244,685.	3.70126	6.27805 × 10^−02^	−87.4438	45.4521	−1.92387	6.53840 × 10^−02^
X1 X7	1	55,311.4	55,311.4	0.836675	3.68755 × 10^−01^	−41.575	45.4521	−0.914699	3.68755 × 10^−01^
X2 X3	1	132,072.	132,072.	1.99781	1.69388 × 10^−01^	−64.2438	45.4521	−1.41344	1.69388 × 10^−01^
X2 X4	1	102,062.	102,062.	1.54385	2.25136 × 10^−01^	56.475	45.4521	1.24252	2.25136 × 10^−01^
X2 X7	1	142,498.	142,498.	2.15551	1.54054 × 10^−01^	76.3713	45.4521	1.46817	1.54054 × 10^−01^
X3 X4	1	38,309.1	38,309.1	0.579498	4.53363 × 10^−01^	−34.6	45.4521	−0.761241	4.53363 × 10^−01^
X3 X7	1	65,685.	65,685.	0.995393	3.28053 × 10^−01^	−45.3062	45.4521	−0.996791	3.28053 × 10^−01^
X4 X7	1	119,805.	119,805.	1.12525	2.89952 × 10^−01^	61.1875	45.4521	1.3462	2.89952 × 10^−01^
X1^2^	1	34,912.8	34,912.8	0.528113	4.73894 × 10^−01^	32.2551	163.653	0.197094	8.45286 × 10^−01^
X2^2^	1	621,655.	621,655.	0.00940354	9.23492 × 10^−01^	−52.2449	163.653	−0.319242	7.52094 × 10^−01^
X3^2^	1	6356.43	6356.43	0.0961513	7.58970 × 10^−01^	28.6051	163.653	0.174971	8.62598 × 10^−01^
X4^2^	1	5326.44	5326.44	0.0805711	7.78773 × 10^−01^	40.6051	163.653	0.248117	8.05993 × 10^−01^
X7^2^	1	934.278	934.278	0.0141325	9.06284 × 10^−01^	19.4551	163.653	0.11888	9.06284 × 10^−01^
Error	26	1.71882 × 10^06^	66,108.6						
Total	46	4.15772 × 10^06^							

DF = Degrees of Freedom; SS = Sum of Squares; MS = Mean Square. *p*-values shown in scientific notation (E). Intercept is labeled ‘1’. Blank cells indicate not applicable. R^2^ = 0.587, Adjusted R^2^ = 0.269.

For the final model: R^2^ = 0.236, Adjusted R^2^ = 0.201.

Standard error prediction at the optimal conditions {−1, 1} of {X2, X3} (and the same for {1, −1}): 72.769.

*ZP*

**Table A4:** ANOVA and Estimated Parameters Table for the response variable *ZP*.

Term	DF	SS	MS	F-Statistic	*p*-Value (ANOVA)	Estimate	Standard Error	t-Statistic	*p*-Value (Coeff)
1						−32.2433	5.07464	−6.35381	9.94643 × 10^−07^
X1	1	2192.03	2192.03	7.51886	1.09020 × 10^−02^	−8.02941	2.92825	−2.74205	1.09020 × 10^−02^
X2	1	829.13	829.13	2.84399	1.03681 × 10^−01^	4.93824	2.92825	1.68641	1.03681 × 10^−01^
X3	1	2431.07	2431.07	8.33878	7.71641 × 10^−03^	8.45588	2.92825	2.88769	7.71641 × 10^−03^
X4	1	2185.61	2185.61	7.49684	1.10053 × 10^−02^	8.01765	2.92825	2.73804	1.10053 × 10^−02^
X7	1	42.2474	42.2474	0.144912	7.06538 × 10^−01^	−1.11471	2.92825	−0.380673	7.06538 × 10^−01^
X1 X2	1	1555.43	1555.43	5.33525	2.90970 × 10^−02^	−6.97188	3.01837	−2.30982	2.90970 × 10^−02^
X1 X3	1	1.08781	1.08781	0.0037313	9.51759 × 10^−01^	−0.184375	3.01837	−0.0610843	9.51759 × 10^−01^
X1 X4	1	632.79	632.79	2.17053	1.52682 × 10^−01^	−4.44687	3.01837	−1.47327	1.52682 × 10^−01^
X1 X7	1	8.30281	8.30281	0.0284794	8.67293 × 10^−01^	−0.509375	3.01837	−0.168758	8.67293 × 10^−01^
X2 X3	1	382.953	382.953	1.31356	2.62190 × 10^−01^	−3.45938	3.01837	−1.14611	2.62190 × 10^−01^
X2 X4	1	478.178	478.178	1.64019	2.11606 × 10^−01^	3.86563	3.01837	1.2807	2.11606 × 10^−01^
X2 X7	1	31.0078	31.0078	0.10636	7.46938 × 10^−01^	−0.984375	3.01837	−0.326128	7.46938 × 10^−01^
X3 X4	1	22.2778	22.2778	0.0764149	7.84401 × 10^−01^	−0.834375	3.01837	−0.276432	7.84401 × 10^−01^
X3 X7	1	18.7578	18.7578	0.064341	8.01757 × 10^−01^	0.765625	3.01837	0.253655	8.01757 × 10^−01^
X4 X7	1	16.9653	16.9653	0.0581926	8.11268 × 10^−01^	0.728125	3.01837	0.241231	8.11268 × 10^−01^
X1^2^	1	2.44127	2.44127	0.00837739	9.27790 × 10^−01^	4.30785	10.8678	0.396368	6.95055 × 10^−01^
X2^2^	1	63.9457	63.9457	0.21934	6.43446 × 10^−01^	−2.74215	10.8678	−0.252318	8.02779 × 10^−01^
X3^2^	1	729.968	729.968	2.50386	1.25658 × 10^−01^	−21.4424	10.8678	−1.973	5.92159 × 10^−02^
X4^2^	1	1399.9	1399.9	4.80178	3.75859 × 10^−02^	24.7079	10.8678	2.27349	3.14926 × 10^−02^
X7^2^	1	108.9	108.9	0.373537	5.46390 × 10^−01^	−6.64215	10.8678	−0.611177	5.46390 × 10^−01^
Error	26	7579.97	291.537						
Total	46	20713.							

DF = Degrees of Freedom; SS = Sum of Squares; MS = Mean Square. *p*-values shown in scientific notation (E). Intercept is labeled ‘1’. Blank cells indicate not applicable. R^2^ = 0.634, Adjusted R^2^ = 0.353.

For the final model: R^2^ = 0.547, Adjusted R^2^ = 0.466.

Standard error prediction at the optimal conditions for the minimum, {0.106, −1, −1, −0.106}, and the maximum, {−1, 1, 1, 1}, of the codified factors X1, X2, X3 y X4, are 9.8259 and 10.687, respectively.

## Appendix C. Relation Between Natural and Codified Factors X_i_

**[ALG] = 0.65 + 0.35 X_1_**

**[CS] = 0.65 + 0.35 X_2_**

**[CaCl_2_] = 0.565 + 0.435 X_3_**

**ALG:CS volume ratio** = 2.50:0.625 + 2.50:0.375 **X_4_**

**ALG:CaCl_2_ volume ratio** = 2.50:0.31 + 2.50:0.15 **X_5_**

**CaCl_2_ flow = 1.25 + 0.75 X_6_**

**CS flow = 1.25 + 0.75 X_7_**
